# Tape‐Assisted Residual Layer‐Free One‐Step Nanoimprinting of High‐Index Hybrid Polymer for Optical Loss‐Suppressed Metasurfaces

**DOI:** 10.1002/advs.202409371

**Published:** 2025-01-07

**Authors:** Yujin Park, Joohoon Kim, Younghwan Yang, Dong Kyo Oh, Hyunjung Kang, Hongyoon Kim, Junsuk Rho

**Affiliations:** ^1^ Department of Mechanical Engineering Pohang University of Science and Technology (POSTECH) Pohang 37673 Republic of Korea; ^2^ Department of Chemical Engineering Pohang University of Science and Technology (POSTECH) Pohang 37673 Republic of Korea; ^3^ Department of Electrical Engineering Pohang University of Science and Technology (POSTECH) Pohang 37673 Republic of Korea; ^4^ POSCO‐POSTECH‐RIST Convergence Research Center for Flat Optics and Metaphotonics Pohang 37673 Republic of Korea; ^5^ National Institute of Nanomaterials Technology (NINT) Pohang 37673 Republic of Korea

**Keywords:** dielectric structural color metausrface, hologram metasurface, nanoparticle‐embedded resin, residual layer removal, tape‐assisted nanoimprint lithography

## Abstract

The commercialization of metasurfaces is crucial for real‐world applications such as wearable sensors, pigment‐free color pixels, and augmented and virtual reality devices. Nanoparticle‐embedded resin‐based nanoimprint lithography (PER‐NIL) has shown itself to be a low‐cost, high‐throughput manufacturing method enabling the replication of high‐index nanostructures. It has been extensively integrated into the fabrication of hologram metasurfaces, metalenses, and sensors due to its procedural simplicity. Most research on PER‐NIL has been limited to exploring appropriate materials to enhance the efficiency of imprinted metasurfaces, but the intrinsic issue of PER‐NIL lies in the high‐index residual layer remaining on the substrate. This high‐index residual layer generates undesired noise, limiting the efficiency and functionality of imprinted metasurfaces. Despite the need for the removal of the residual layer, it has never been experimentally achieved owing to the different etching rates between the nanoparticles and resin. Here, a new methodology named tape‐assisted PER‐NIL is proposed, achieving one‐step removal of the residual layer using a tape. This novel method enables the replication of residual layer‐free, high‐index metasurfaces. As a result, imprinted residual layer‐free metasurfaces prove their potential in high‐purity dielectric structural colorations by achieving a sharp reflectance peak unattainable with conventional NIL, and in vivid hologram metasurfaces by covering a full 2π phase without unwanted scattering.

## Graphical abstract

A new fabrication methodology for residual layer‐free, high‐index, one‐step replicable metasurfaces is proposed. Adhesive tape is employed to eliminate the high‐index residual layer that has generated optical noise so far. Printable structural colors with sharp single peaks and replicable hologram metasurfaces of high quality, both unattainable with conventional imprinting, are demonstrated with these residual layer‐free metasurfaces.

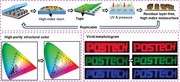

## 1 Introduction

Metasurfaces, comprising subwavelength structures called meta‐atoms, permit the precise modulation of light wavefronts with their compact form factor.^[^ ^1^ ^]^ They have been extensively studied as alternatives to overcome the limitations of conventional bulky optical systems, finding applications in various fields such as thin lenses,^[^ ^2^ ^]^ holograms,^[^ ^3^ ^]^ color filers,^[^ ^4^ ^]^ and other optical systems.^[^ ^5^ ^]^ However, manufacturing constraints, including high cost and low throughput, have impeded the widespread use and applications of metasurfaces.^[^ ^6^ ^]^ To address these issues, nanoimprint lithography (NIL) has been employed for scalable manufacturing of metasurfaces.^[^ ^7^ ^]^ In the conventional NIL process, imprinted commercial resin is unsuitable as the final structure because of its low refractive index (≈1.5). Instead, an imprinted structure is used as an intermediate medium, requiring secondary operations such as etching, deposition,^[^ ^8^ ^]^ and annealing.^[^ ^9^ ^]^ To simplify the fabrication process, a high‐index printable material called nanoparticle‐embedded resin (PER) has been proposed. The PER, prepared by dispersing dielectric nanoparticles into resin, offers a promising solution for low‐cost and high‐throughput single‐step manufacturing of metasurfaces.^[^ ^10^ ^]^

However, PER‐based NIL (PER‐NIL) is limited by the formation of an undesirable residual layer on the substrate. A high‐index residual layer composed of dielectric nanoparticles remains on the substrate after the imprinting process. This high‐index residual layer results in unexpected optical properties for both Mie‐resonance and phase modulation metasurfaces. In Mie‐resonance structural colors fabricated by PER‐NIL, achieving high color purity has been challenging.^[^ ^10c^ ^]^ Ideally, Mie‐resonance occurs only in the designed meta‐atoms, resulting in a sharp peak at the target wavelength and thus, high color purity. However, a high‐index residual layer creates additional unintended resonance modes. These extra modes create unwanted peaks, broadening the spectral responses, and consequently reducing the purity of the structural color.^[^ ^10c^ ^]^ In phase modulation metasurfaces using geometric or propagation phases, dielectric nanoparticles from the residual layer cause light scattering, resulting in undesired noise and a reduction in efficiency.^[^ ^10a,b,d–g^ ^]^ Moreover, controlling the residual layer thickness and uniformity is difficult, particularly at wafer scales, owing to the PER particle size of ≈30 nm. This lack of precise control significantly impacts conversion efficiency, resulting in inconsistent performance and decreased overall efficiency.^[^ ^10d^ ^]^

Eliminating the residual layer in PER‐based metasurfaces is crucial; however, this has remained an elusive goal because of its highly challenging nature. Residual layers consisting of conventional imprint resin can be dry etched, whereas those consisting of PER are difficult to etch owing to the different etching rates of the matrix resin and nanoparticles. Therefore, despite their significance, residual layer‐free PER metasurfaces have not been achieved yet.^[^ ^5^, ^10^, ^11^ ^]^

Here, we achieved replication of residual layer‐free, high‐index meta‐atoms in a single step using novel tape‐assisted PER‐NIL. PER enables the duplication of high‐index meta‐atoms without additional needs to increase the refractive index, and the tape enables one‐step removal of residual layers without any operational equipment. To establish a stable transfer protocol, we determine the optimal PER concentration and the most suitable adhesive tape for the residual layer removal process. Successful removal of the residual layer is confirmed through scanning electron microscopy (SEM) images and energy‐dispersive X‐ray spectroscopy (EDS) analyses. As a proof of concept, our tape‐assisted PER‐NIL is used to enhance the performance in both structural color metasurfaces (amplitude modulation) and hologram metasurfaces (phase modulation). The replicated structural color metasurface exhibits a sharp single reflectance peak, which is challenging to achieve using conventional NIL. In addition, the replicated hologram metasurface displays a clear holographic image without noise from unwanted diffraction, showing a marked improvement over conventionally imprinted hologram metasurfaces. The removal of residual layer, which is a significant obstacle to achieving high‐performance, is expected to facilitate the commercialization of metasurfaces, particularly when considering the mass productivity and cost‐effectiveness of PER‐NIL.

## 2 Results

### 2.1 Fabrication of Residual Layer‐Free, One‐Step Printable Metasurfaces

**Figure 1:**
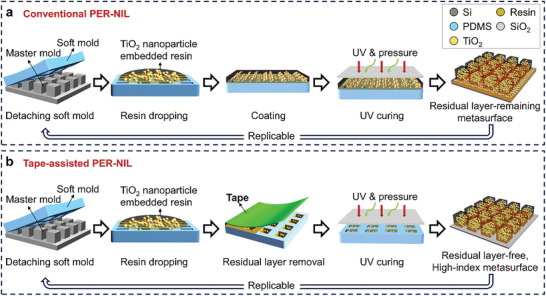
Tape‐assisted nanoparticle‐embedded resin (PER) based nanoimprint lithography (NIL) (PER‐NIL) for one‐step printable, residual layer‐free metasurfaces. a) Schematic of conventional PER‐NIL technique. b) Schematic of tape‐assisted PER‐NIL, which enables duplication of residual layer‐free meta‐atoms.

**Figure 2:**
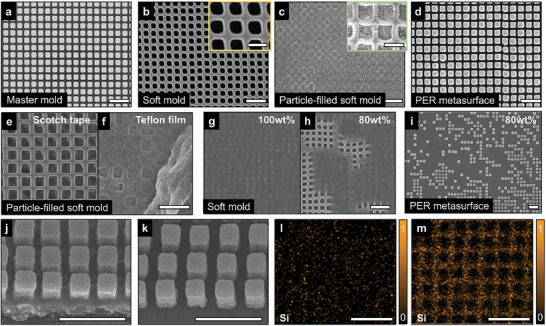
Fabrication results of residual layer‐free metasurfaces using the tape‐assisted PER‐NIL. Scanning electron microscope (SEM) images: a) master mold, b) soft mold, c) soft mold filled with PER after removing residual layer, and d) imprinted residual layer‐free metasurface. Nanoparticle‐filled soft mold with residual layer removed using e) scotch tape, and f) teflon film. Soft mold fabricated by toluene‐diluted polydimethylsiloxane (PDMS) with weight ratios of g) 100%, and h) 80%. i) Imprinted PER metasurface with 80% TiO_2_ particle weight ratio. 15° tilted SEM images: j) residual layer‐remaining metasurface using conventional PER‐NIL and k) residual layer‐free metasurface fabricated by tape‐assisted PER‐NIL process. Energy dispersive X‐ray spectroscopy (EDS) analyses of Si component of l) conventionally imprinted metasurface with residual layer, and m) residual layer‐free metasurface. The substrate material is SiO_2_. All scale bars: 1 µm. Inset scale bars: 0.5 µm.

The duplication of high‐index and residual layer‐free meta‐atoms is achieved by the development of a tape‐assisted PER‐NIL as shown in **Figures** **1** and **2**a–d. PER, a high‐index imprintable material, is prepared by mixing high‐index nanoparticles with an ultraviolet (UV) curable resin. Nanoparticles serve to increase the effective index of PER, complementing the low‐index (≈1.5) UV‐curable resin, whereas the resin acts as a cross‐linker for nanoparticles, enabling the printing of meta‐atoms. In this study, TiO_2_ nanoparticle‐embedded resin (TiO_2_‐PER) is utilized to demonstrate its application in the visible wavelength range.

Residual layer‐free meta‐atoms can be transferred by simply peeling off the residual layer of nanoparticles from a soft mold. Nanoparticles are removed before crosslinking occurs between particles by UV curing, preserving the particles inside the meta‐atoms. The key to achieving residual layer‐free metasurface is to selectively peel off the residual layer without affecting the meta‐atoms.^[^ ^12^ ^]^ Therefore, various tapes with different adhesion levels are tested to ensure uniform removal of only the residual layer and not the meta‐atoms. If the adhesion is too high, the meta‐atoms are partially peeled off (Figure 2e); if it is too low, the residual layer is not uniformly removed (Figure 2f). Therefore, polyethylene tape is selected as the optimal tape for uniformly peeling off the residual layer (Figure S1, Supporting Information). To evaluate the performance of the polyethylene tape, SEM images of the fabricated samples are captured multiple times over a wide area, confirming that the tape enables the uniform removal of the residual layer (Figure S2, Supporting Information). The additional process details are provided in the Experimental section.

Meanwhile, the PER weight ratio is optimized to ensure both high transferability of residual layer‐removed meta‐atoms and highly‐efficient, imprinted metasurfaces. The transferability of meta‐atoms is strongly influenced by the PER weight ratio, as the resin acts as a cross‐linker between nanoparticles, enabling the replication of high‐index structures. Similarly, the efficiency of the metasurface is highly dependent on the PER weight ratio, as the nanoparticles contribute to increasing the effective refractive index (Figure S3, Supporting Information), improving the conversion efficiency in hologram metasurfaces (Figure S4, Supporting Information) and enhancing light confinement in structural color metasurfaces (Figure S5, Supporting Information). Therefore, the PER weight ratio must be carefully balanced to achieve both optimal transferability and high efficiency. Since efficient light control can be achieved starting from 60 wt.% (Figures S3–S5, Supporting Information), weight ratios of 60% and 80% are tested to ensure the stable transferability of the meta‐atoms. The 60 wt.% PER shows high transferability of meta‐atoms onto the substrate (Figure 2d), whereas the 80 wt.% PER exhibits poor transfer quality of meta‐atoms due to insufficient resin for crosslinking nanoparticles (Figure 2i). Therefore, the PER is synthesized by dispersing TiO_2_ nanoparticles at a weight ratio of 60% into a UV‐curable resin (Figure S6, Supporting Information). In addition, the size of the TiO_2_ nanoparticles is carefully chosen. As the nanoparticle size increases, beam scattering in the residual layer becomes more severe (Figure S7, Supporting Information). Therefore, the minimum synthesizable size of 30 nm is primarily used to minimize the scattering effect. However, even at a particle size of 30 nm, the scattering cannot be ignored, highlighting the novelty of removing the residual layer.

Additionally, we prepare a soft mold capable of replicating nanoscale patterns from the master mold using toluene‐diluted polydimethylsiloxane (PDMS). For the soft mold to possess reusability and replicability, both low surface tension and flexibility are required. Although PDMS is suitable for soft molds because of its low surface tension and flexibility,^[^ ^13^ ^]^ replicating nanoscale structures from a master mold is challenging because of its high viscosity. Thus, we utilize toluene‐diluted PDMS to adjust the viscosity of PDMS. PDMS solutions with weight ratios of 100%, 80%, and 60% are tested to determine their ability to replicate meta‐atoms from the master mold. The results show that 60 wt.% diluted PDMS is sufficient to duplicate the nanoscale structures (Figure 2g,h; Figure S8, Supporting Information). Moreover, the successful infiltration of PER and the uniformity of nanoparticles within the nanostructures are evident from the SEM images of the particle‐filled soft mold (Figure 1c).

SEM images and EDS analyses are utilized to confirm the successful removal of the residual layer. In Figure 2k, the residual layer‐removed metasurface is verified through SEM images, whereas conventionally imprinted metasurface has a residual layer of ≈150 nm, as shown in Figure 2j. In addition, an EDS analysis is conducted with and without a residual layer (Figure 2l,m; Figure S9, Supporting Information). The removal of the residual layer is demonstrated by detecting substrate element Si, as the substrate is exposed in regions without a residual layer. Consequently, the Si signal appears strongly in the residual layer‐free regions (Figure 2m). In the sample with the residual layer, the Si signal becomes significantly weak, which is attributed to the coverage of the entire sample with the TiO_2_ residual layer (Figure 2l). Therefore, the Ti element shows a much higher atomic percentage in the sample with a residual layer (39.05%) than in the sample without a residual layer (20.80%) (Figure S9, Supporting Information).

### 2.2 Residual‐Free Imprinted Dielectric Structural Color Metasurface: Achieving a Sharp Single Reflectance Peak

Structural color metasurfaces with a sharp single reflectance peak are achieved using tape‐assisted PER‐NIL. To design a high‐purity structural color metasurface, the phase difference and amplitude ratio of light reflected from the PER thin‐film are measured using ellipsometry. Subsequently, the optical properties of 60 wt.% TiO_2_‐PER are determined by fitting the Cauchy dispersion model to the measured optical properties (Figure S10, Supporting Information). The refractive indices are 1.96, 1.89, and 1.85, and the extinction coefficients are 0.0026, 0.00059, and 0.00015 at wavelengths of 450, 532, and 635 nm, making the TiO_2_‐PER suitable for use in the visible region. Subsequently, a rectangular shape is chosen for the meta‐atoms, characterized by the period (*p*), gap (*g*), thickness (*t*), and width (*w*), where *w* = *p – g*. The residual layer is designed as a film with a thickness of *t_residual_*.

**Figure 3:**
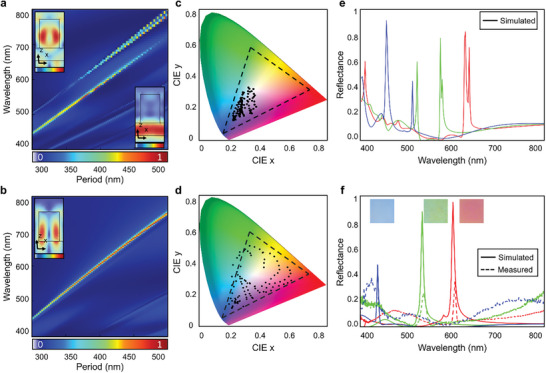
Imprinted residual layer‐free structural color metasurfaces. a,b) Calculated reflectance spectrum for different periods (*p*) of metasurface with a gap (*g*) = 120 nm and meta‐atom thickness (*t*) = 340 nm with a residual layer thickness (*t_residual_*) of 150 nm (a) and without residual layer (b). Inset: corresponding electromagnetic field distribution at resonance wavelengths. c,d) Simulated color gamut on CIE 1931 map of metasurfaces with varying *p* and *g* at *t* = 340 nm with *t_residual_* = 150 nm (c) and without residual layer (d). e) Simulated reflectance spectra for blue (*p = *310 nm), green (*p = *360 nm), and red (*p = *400 nm) with *t_residual_* = 150 nm. f) Simulated (solid line) and measured (dashed line) reflectance spectra for blue (*p = *310 nm), green (*p = *360 nm), and red (*p = *400 nm) without residual layer. Inset: Captured optical microscope images of imprinted structural color metasurfaces.

The reflectance of the metasurface with a residual layer is plotted by varying *p* from 300 to 500 nm with a constant *t* of 340 nm, *g* of 120 nm, and *t_residual_* of 150 nm to investigate the optical characteristics of the conventionally imprinted structural color metasurface (**Figure** **3**a). Then, the electromagnetic field distributions are analyzed at the resonance wavelengths, where a confined distribution is observed not only within the designed meta‐atoms but also in the high‐index residual layer^[^ ^10c^ ^]^ (inset of Figure 3a). As a result, two reflectance peaks, one attributed to the meta‐atom and the other to the residual layer are observed across the wavelength range from 400 to 800 nm (Figure 3a).

However, to obtain a high‐purity color, a sharp single reflectance peak is required. Therefore, the reflectance is plotted for the residual layer‐free structural color metasurface by varying *p* from 300 to 500 nm with a constant *t* of 340 nm and *g* of 120 nm. As a result, a sharp single peak appears in the residual layer‐free metasurfaces, with the inset indicating that the single reflectance peak originated solely from the designed meta‐atom (Figure 3b). The resulting color gamut of our structural color metasurfaces are plotted by sweeping *p* from 280 to 480 nm and *g* from 60 to 140 nm with a constant *t* of 340 nm and *t_residual_* of 150 nm. A wide color gamut of the residual layer‐free structural color metasurface is confirmed, which covers most of the color gamut of the sRGB, represented by a dashed line (Figure 3c,d).

In Figure 3e, blue, green, and red are designed with unit sizes *p = *310, 360, and 400 nm with *t = *340 nm, *g = *120 nm, *and t_residual_ = *150 nm, respectively. Structural color metasurfaces without a residual layer are also designed for blue, green, and red with unit sizes *p = *300, 360, and 400 nm*, g = *130, 130, and 120 nm, respectively, and the same *t = *330 nm. To experimentally compare the reflectance spectra, structural color metasurfaces with and without a residual layer are fabricated (Figure S11, Supporting Information), and their reflectance spectra are measured (Figure S12, Supporting Information). For metasurfaces with a residual layer, the reflectance peak position deviates from the simulated spectra due to difficulties in adjusting the residual layer thickness to the designed value; however, two distinct reflectance peaks, as predicted in the simulation, are still observed (Figure S13, Supporting Information). In contrast, the residual layer‐free metasurfaces exhibit single reflectance peaks, confirming the agreement between the measured and simulated spectra (Figure 3f). The slightly lower measured reflectance is attributed to limitations in the measurement setup, particularly the oblique incidence of light due to the numerical aperture (NA) of the objective lens.^[^ ^4e^ ^]^ In addition, fabrication imperfections, such as morphological irregularities and thickness variations, may contribute to the reduced reflectance intensity.

Another limitation of conventional imprinted structural color metasurface is the uncontrollable residual layer thickness, which leads to uneven color, as shown in Figure S14 (Supporting Information). The speckled color occurs because the reflectance spectrum changes not only depending on the meta‐atom but also depending on the residual layer thickness significantly. Therefore, our residual layer‐free structural color metasurface is more robust to fabrication errors, enabling uniform coloration. Additionally, the residual layer‐free sample is successfully fabricated in a 1 cm × 1 cm area, featuring a 3 × 3 array of 3 mm × 3 mm patterns, demonstrating the potential of the tape‐assisted PER‐NIL method for wafer‐scale scalability (Figure S15, Supporting Information).

### 2.3 Residual‐Free Imprinted Hologram Metasurfaces: Suppressing Noises in Holographic Images

A printable hologram metasurface displaying clear images across a broad range of wavelengths is obtained using tape‐assisted PER‐NIL. A full 2π phase modulation is achieved for high‐quality hologram metasurface by rotating angles of anisotropic meta‐atoms, known as the Pancharatnam‐Berry (PB) phase.^[^ ^10^, ^14^ ^]^ For rotated meta‐atoms, the conversion efficiency (*CE*) can be calculated as follows:

(1) $$E_{T}=\frac{t_{xx}+t_{yy}}{2}\;E_{co-pol}+\frac{t_{xx}-t_{yy}}{2}e^{i2\theta }E_{cross-pol}$$

where *E_T_* is the electric fields of the transmitted light; *E*_*co*−*pol*_, and *E*_*cross*−*pol*_ are the co‐ and cross‐polarized components, respectively. In Equation (1), *E*_*co*−*pol*_ causes zero‐order diffraction while the phase of *E*_*cross*−*pol*_ is retarded by twice the rotating angle (*θ*) of meta‐atoms. Therefore, the amplitude of the *E*_*cross*−*pol*_ should be maximized to obtain a high‐quality holographic image, known as the *CE*, which can be obtained as follows:

(2) $$CE={\left|\frac{t_{xx}-t_{yy}}{2}\right|}^{2}$$

According to Equation (2), meta‐atoms with the highest *CE* are calculated by varying the lengths (*l*) from 250 to 400 nm and widths (*w*) from 50 to 200 nm, with a constant height (*h*) at 1,000 nm, using a rigorous coupled‐wave analysis (RCWA). A structure with *l* = 400 nm and *w* = 104 nm, which exhibits the highest *CE* of 88%, is selected as the optimal meta‐atom. Subsequently, the Gerchberg‐Saxton algorithm is utilized to obtain a phase map of the holographic image. We design a phase map in eight steps and modulate the phase using an optimized meta‐atom (Figure S16, Supporting Information).

**Figure 4:**
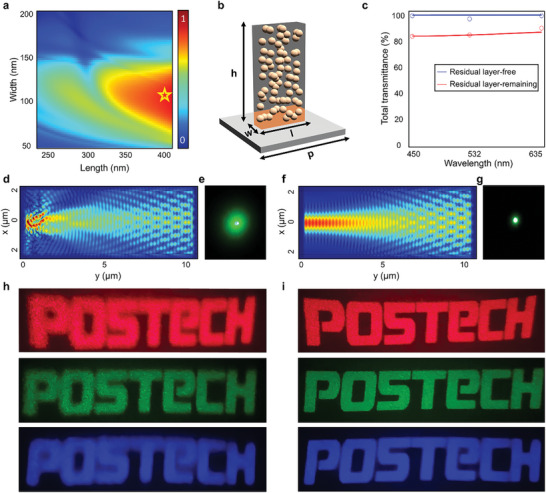
Imprinted residual layer‐free hologram metasurfaces. a) Simulated conversion efficiency (*CE*) of nanostructure with fixed thickness (*h*) = 1000 nm and period (*p*) = 450 nm at wavelength (*λ*) = 532 nm. b) Schematic of imprinted meta‐atom. c) Graph of light transmittance of a substrate with and without a residual layer as a function of wavelength. The red line represents SiO_2_ substrate with *t_residual_* = 70 nm; the blue line represents one without a residual layer. The line represents the simulated data, and the dot represents the measured data. d) The simulated result and e) the captured image of beam scattering through a residual layer of *t_residual_* = 70 nm. f) The simulated result and g) the captured image of beam scattering through a bare substrate without a residual layer. h,i) Captured holographic images of metasurfaces (h) with residual layer and (i) without residual layer.

To evaluate the quality of the holographic images, two types of hologram metasurfaces are prepared: one is a residual layer‐free sample (Figure S17, Supporting Information) and the other has a residual layer of ≈70 nm. Then, the holographic images are captured at wavelengths of 450, 532, and 635 nm respectively (Figure S18, Supporting Information). As shown in **Figure** **4**h,i, the residual layer‐free hologram metasurface shows clearer images over a broadband wavelength range of 450 to 635 nm, demonstrating broadband operation. In addition, normalized intensity profiles are analyzed for samples with and without a residual layer. The blurring effect on the edges of the letters owing to the residual layer is verified through the intensity profile (Figure S19, Supporting Information).

Low‐quality hologram metasurfaces with residual layer can be explained by two main factors: (i) reduced transmittance of light passing through the residual layer, and (ii) scattering of light passing through the residual layer. The simulated and experimental results for the first factor are shown in Figure 4c, which demonstrates the decreased transmission in the residual layer. Light transmittance is critical for achieving high efficiency of transmissive metasurfaces because the *CE* of the metasurface is determined by the light transmittance. Therefore, a reduction in *CE* ^[^ ^10d^ ^]^ of metasurfaces, attributed to the reduced transmittance, is confirmed in Figure S20 (Supporting Information). The second factor involves beam scattering through the layer because the residual layer consists of high‐index nanoparticles. The simulated result is shown in Figure 4d,f, which is further confirmed experimentally by illuminating light through a bare substrate and one with a residual layer. As a result, beam scattering is observed when passing through the residual layer (Figure 4e), while light passing only through the bare substrate does not scatter significantly (Figure 4g).

## 3 Conclusion

PER‐NIL has been extensively used in various optical fields, offering single‐step fabrication, scalable manufacturing, and cost‐effective production of metasurfaces. However, its inherent limitation, a high‐index residual layer, induces undesirable optical losses, limiting the performance and application of imprinted metasurfaces. The residual layer is not only challenging to remove through plasma etching but also to control its thickness using a spin‐coating method (Figure S21, Supporting Information). Moreover, even with minimal residual layer thickness or particle size, unwanted optical losses still occur (Figures S7 and S22, Supporting Information).

We present a novel tape‐assisted PER‐NIL method for eliminating the high‐index residual layer. The key innovation of this approach lies in the use of tape, which effectively removes the residual layer in a simple but powerful manner, eliminating the need for any operational equipment such as reactive ion etching. As a result, we successfully demonstrate the replication of residual layer‐free, high‐index meta‐atoms. Furthermore, optimized weight ratios of PDMS and PER, along with tape adhesion tests, ensure both high transferability (Figure S2, Supporting Information) and consistent thickness of transferred meta‐atoms (Figure S23, Supporting Information). Tape‐assisted PER‐NIL also shows its potential applications in the fabrication of dielectric structural color metasurfaces and hologram metasurfaces. Imprinted structural color metasurfaces exhibit a sharp single reflectance peak which is unattainable with conventional NIL, and imprinted hologram metasurfaces show high‐quality images across a broad wavelength range compared to conventional methods.

The replication of residual layer‐free, high‐index meta‐atoms is expected to be adopted in various applications, such as color pixels, augmented reality/virtual reality displays, and high‐performance metalenses considering the benefits of cost‐effectiveness, various substrate selectivities, and mass productivity of the proposed approach. Furthermore, the physical peeling method employed to remove the residual layer operates effectively not only for TiO_2_ but also for various other materials, demonstrating the broad applicability of the tape‐assisted PER‐NIL method.

## 4 Experimental Section

### Master Mold Fabrication

Positive‐tone photoresist (495 PMMA A6, MicroChem & 950 PMMA A2, MicroChem) was spin‐coated onto a silicon substrate at 2000 rpm and subsequently cured at 180 °C for ≈5 min. The pattern was transferred onto the photoresists using the electron beam lithography (EBL) (ELS‐7800, Elionix), and the exposed sample was developed in the solution (MIBK: IPA = 1:3, MicroChem) at 0 °C for 10 min. A chromium (Cr) layer, 50 nm thick, was deposited on the sample by electron beam evaporation (KVT, KVE‐ENS4004). The deposited Cr layer was utilized as an etching mask to vertically etch the substrate. A plasma etching (DMS, Si/metal hybrid etcher) was conducted to vertically etch the Si substrate, and the Cr etchant (ETCR300) was used to remove the remaining Cr layer. Subsequently, the fabricated master mold was vapor‐coated [(tridecafluoro‐1,1,2,2‐tetrahydrooctyl)trichlorosilane] at 130°C for 5 min twice to facilitate the demolding of soft mold from the master mold.

### Soft Mold Fabrication

A mixture of PDMS (Sylgard 184 A, Dow Corning) and its curing agent (Sylgard 184 B, Dow Corning) in a weight ratio of 10:1 was diluted in toluene at a 60% weight ratio. The PDMS solution was dropped onto the master mold, spin‐coated at 2000 rpm for 60 s, and subsequently cured at 70 °C for 1 h. Afterward, the mixture of PDMS and curing agent was poured onto the cured PDMS thin layer and cured at 70 °C for 2 h. The baked soft mold and the master mold were carefully detached. The detached soft mold was vapor‐coated with a fluorosurfactant, [(tridecafluoro‐1,1,2,2‐tetrahydrooctyl)trichlorosilane], at 130 °C for 5 min to reduce the surface tension of the mold.

### PER Synthesis

The PER was made by dispersing TiO_2_ nanoparticle‐dispersed MIBK (DT‐TIOA‐30MIBK (N30), Ditto Technology) into a mixture of a monomer (dipentaerythritol penta‐/hexa‐acrylate, Sigma–Aldrich), photo‐initiator (1‐Hydroxycyclohexyl phenyl ketone, Sigma–Aldrich) and MIBK. To fabricate 60 wt.% PER, 1.7 wt.% TiO_2_ NPs, 0.7 wt.% monomer and 0.3 wt.% photo‐initiator were mixed.

### Residual Layer Removal

The tape was attached and detached from the PER‐filled soft mold to remove the residual layer uniformly, and this work was repeated until the soft mold appeared clean. Scotch tape (7012785392, 3 m), polyethylene tape (7000001163, 3 m), and a Teflon film (K45292824, TGK) were prepared for the optimal tape test.

### Residual Layer‐Free Metasurface Imprinting

The pre‐cleaned SiO₂ substrate was treated with O₂ plasma (CUTE‐1MPR, Femto Science Inc.) for 5 min at 100 W and 100 sccm gas flow rate. Then, polymethyl methacrylate resin(495 PMMA A2, MicroChem) was spin‐coated at 2000 rpm for 1 min to increase the surface tension of the substrate, improving adhesion between the meta‐atoms and the substrate. The PMMA‐coated substrate was then baked at 180 °C for 10 min to cure the PMMA layer. After curing, the residual layer‐free, PER‐filled soft mold was pressed onto the substrate, followed by UV illumination at 5 bar for 15 min. Finally, the soft mold was gently peeled off, leaving behind high‐index, residual layer‐free nanostructures on the substrate.

### Reflectance Measurement Setup

A Xenon arc lamp (66907‐150XF‐R1, Newport) was used as the light source and was incident on an objective lens (LMPLFLN 4X, Olympus). The focused light was incident on the sample and the reflected light was collected using an objective lens. The reflected light was split and directed to a fiber‐coupled spectrometer (CCS100, Thorlabs) using a beam splitter (TFA‐30C05‐10, SIGMAKOKI).

### Hologram Measurements Setup

Lasers with 450, 532, and 635 nm (450, 532, and 635 nm diode‐pumped solid‐state laser, Thorlabs) were passed through several optical components to generate the right circularly polarized (RCP) light. This setup included a linear polarizer (Ø ½ in. unmounted linear polarizers, Thorlabs), a half‐wave plate (Ø ½ in. mounted achromatic half‐wave plates, Thorlabs), and a quarter‐wave plate (Ø ½ in. mounted achromatic quarter‐wave plates; Thorlabs). A pinhole with 500 µm diameter (P500HD – Ø1/2 in. (12.7 mm) Mounted Pinhole, Thorlabs) was used to filter the unwanted light. A photodiode power sensor (S120C, Thorlabs) was used to measure the light intensity, which was connected to a compact power and energy meter console (PM100D, Thorlabs).

### Numerical Simulation

The CIE1931 map was plotted using data simulated by rigorous coupled‐wave analysis (RCWA). The spectrum and its color were simulated using the lumerical finite‐difference time‐domain method (FDTD Lumerical). The electrical fields of the structural color metasurfaces were simulated with the finite element method (FEM) (COMSOL Multiphysics 6.5).

## Conflict of Interest

The authors declare no conflict of interest.

## Author Contributions

J.R. and Y.P. conceived the idea and initiated the project. Y.P. and J.K. simulated the optical performances of residual layer‐free and residual layer‐remaining metasurfaces. Y.P. and D.K.O. performed the nanoimprinting. J.K. and H.J.K. fabricated master molds of structural color metasurfaces and hologram metasurfaces. Y.P., J.K., and Y.Y. performed the experimental characterization and data analysis. Y.P. mainly wrote the manuscript. J.R., J.K., and Y.Y. revised the manuscript. All the authors confirmed the final manuscript. J.R. guided the entire project.

## Supporting information



Supporting Information

## Data Availability

The data that support the findings of this study are available from the corresponding author upon reasonable request.
