# Supplementary material for: Tape‐Assisted Residual Layer‐Free One‐Step Nanoimprinting of High‐Index Hybrid Polymer for Optical Loss‐Suppressed Metasurfaces
Source: Adv Sci (Weinh). 2025 Jan 7;12(10):2409371. doi: 10.1002/advs.202409371 (PMC11905001; doi:10.1002/advs.202409371)
Supplement: Supplementary file 1 — Supporting Information [file ADVS-12-2409371-s001.docx]

Supporting Information

Tape-Assisted Residual Layer-Free One-Step Nanoimprinting of High-Index Hybrid Polymer for Optical Loss-Suppressed Metasurfaces

Yujin Park^a^, Joohoon Kim^a^, Younghwan Yang^a^, Dong Kyo Oh^a^, Hyunjung Kang^a^, Hongyoon Kim^a^, Junsuk Rho*^a,b,c,d,e^

**
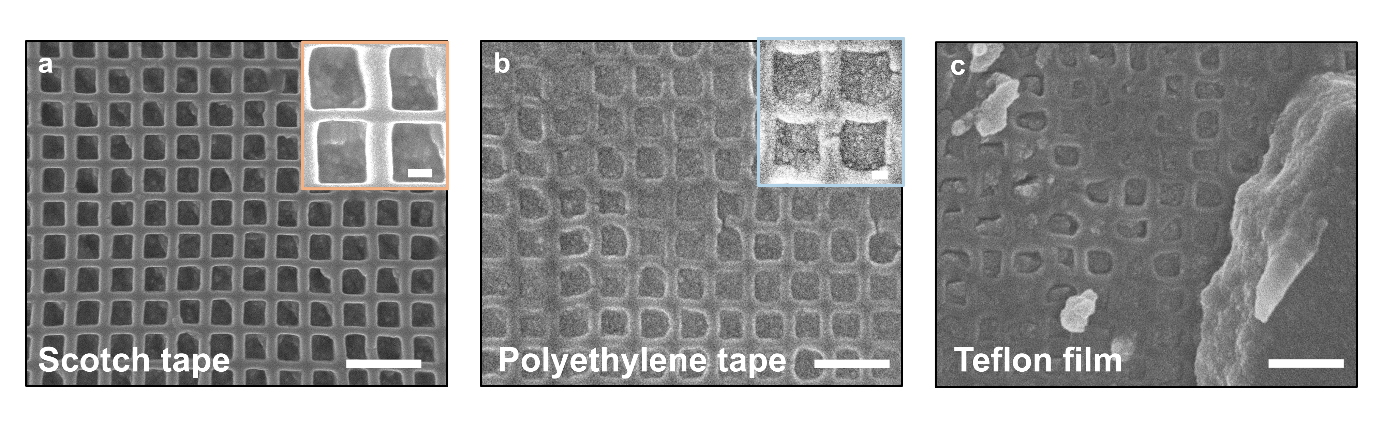
 Figure S1. Adhesion tests of various tapes.** SEM images of soft molds where the residual layers are removed by using **(a)** scotch tape (7012785392, 3M), **(b)** polyethylene tape (7000001163, 3M), and **(c)** teflon film (K45292824, TGK). All scale bars: 1 µm.

**
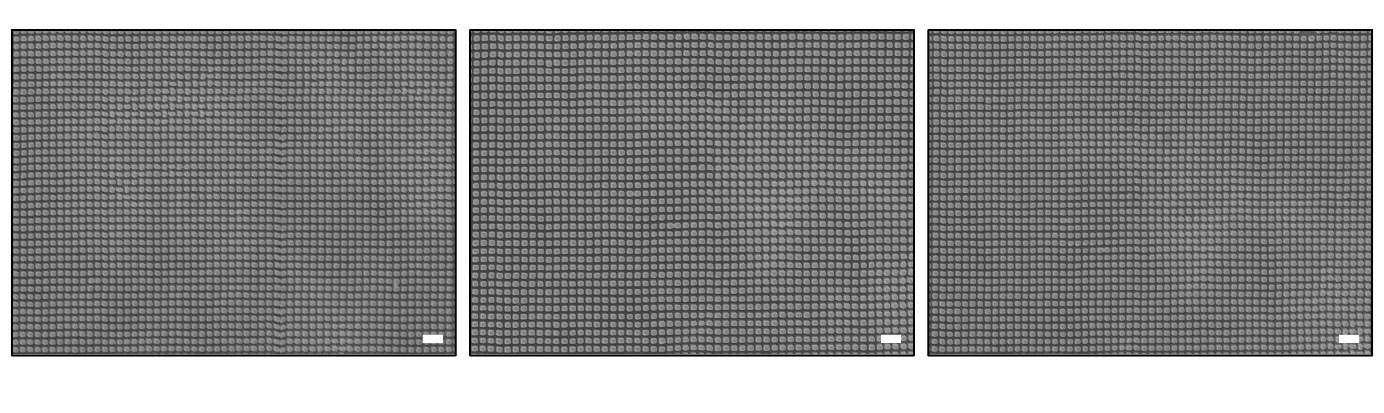
**

**Figure S2.** **SEM images of imprinted metasurfaces where the residual layers are removed by Polyethylene tape.** Three images are captured from different areas. All scale bars: 1 µm.

**
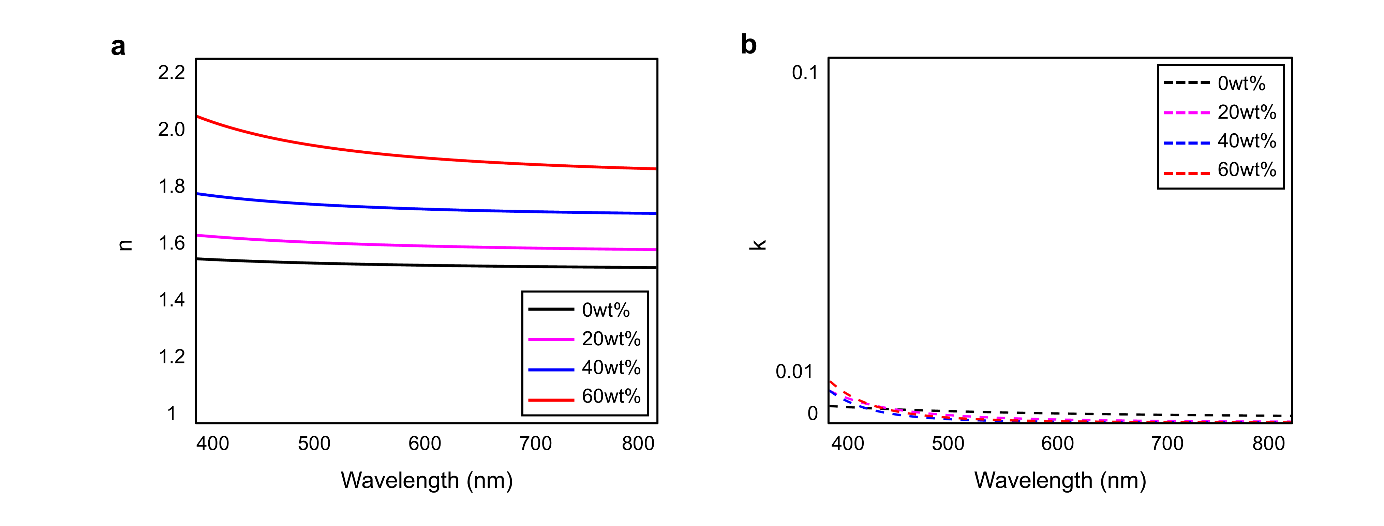
**

**Figure S3. Optical properties of particle-embedded resins (PERs) with various nanoparticle weight ratios.** (a) Measured refractive index (*n*) at different weight ratios. (b) Measured extinction coefficient (*k*) at different weight ratios.

**
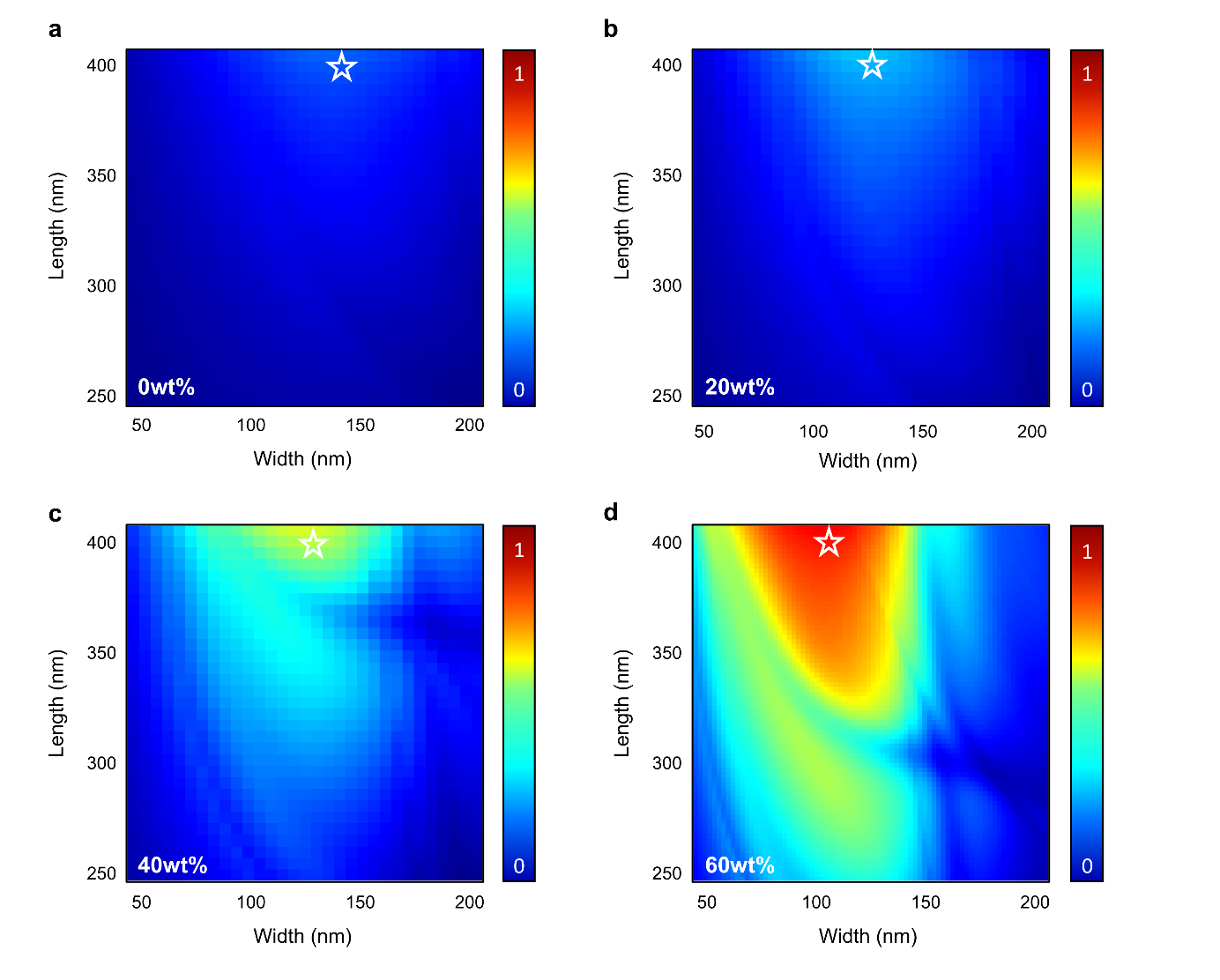
**

**Figure S4. Simulated conversion efficiencies (*CE*) of metasurfaces with different weight ratios of PER.** The Meta-atoms have a fixed period (*p*) = 450 nm, thickness (*h*) = 1000 nm, and wavelength (*λ*) = 532 nm, with nanoparticle weight ratios of **(a)** 0 wt%, **(b)** 20 wt%, **(c)** 40 wt%, and **(d)** 60 wt%. The maximum *CE* for each plot is **(a)** 21.8%, **(b)** 31.3%, **(c)** 58.4%, and **(d)** 88%.

**
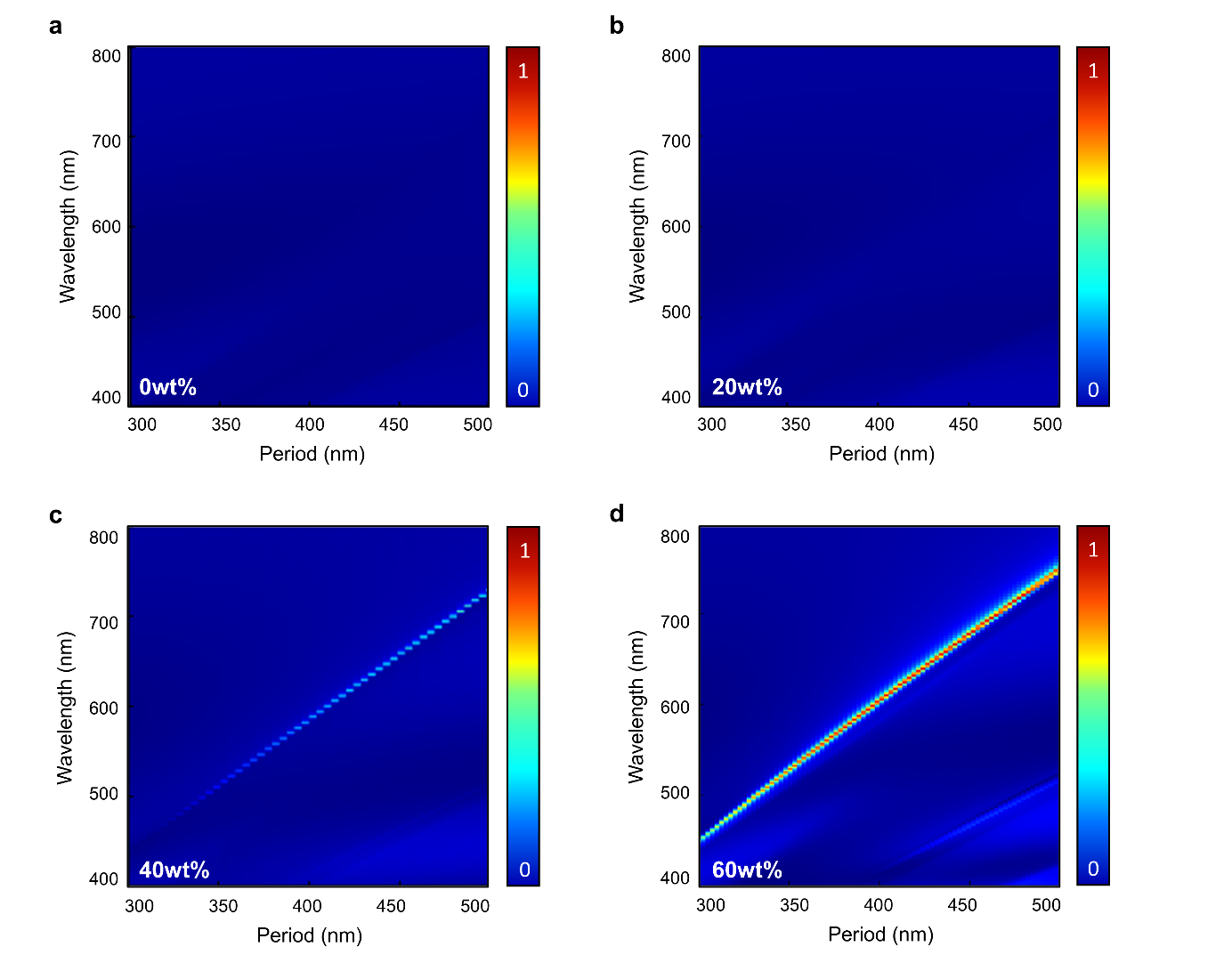
**

**Figure S5. Calculated reflectance spectra of imprinted structural color metasurfaces with varying weight ratios of PER.** Meta-atoms have a fixed gap (*g*) = 120 nm, thickness (*t*) = 340 nm, with nanoparticle weight ratios of (a) 0 wt%, (b) 20 wt%, (c) 40 wt%, and (d) 60 wt%.

**
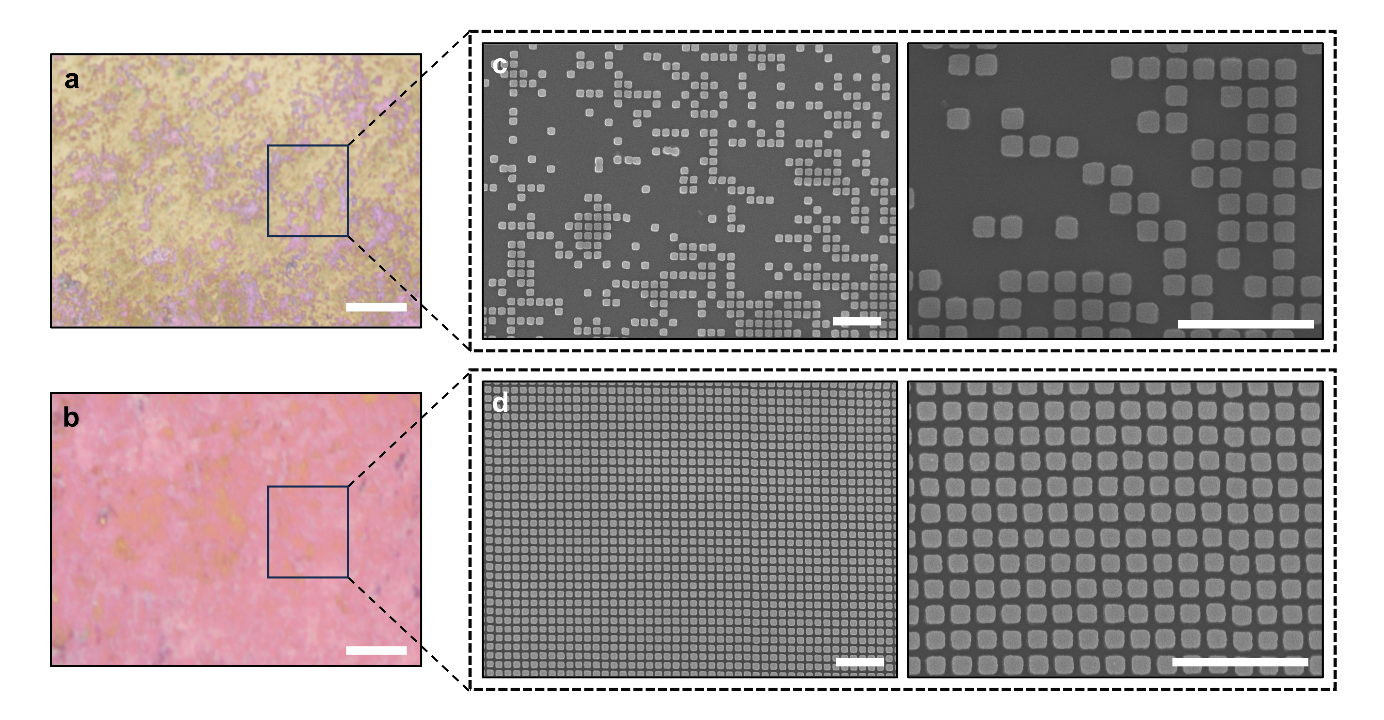
**

**Figure S6. Transferability tests using different weight ratios of PER.** Optical microscope (OM) images of an imprinted residual layer-free structural color metasurfaces with TiO_2_ particle weight ratios of **(a)** 80%, and **(b)** 60%. Sclae bars: 100 µm. SEM images of the imprinted residual layer-free structural color metasurfaces with TiO_2_ particle weight ratios of **(c)** 80%, and **(d)** 60%. Scale bars: 2 µm.


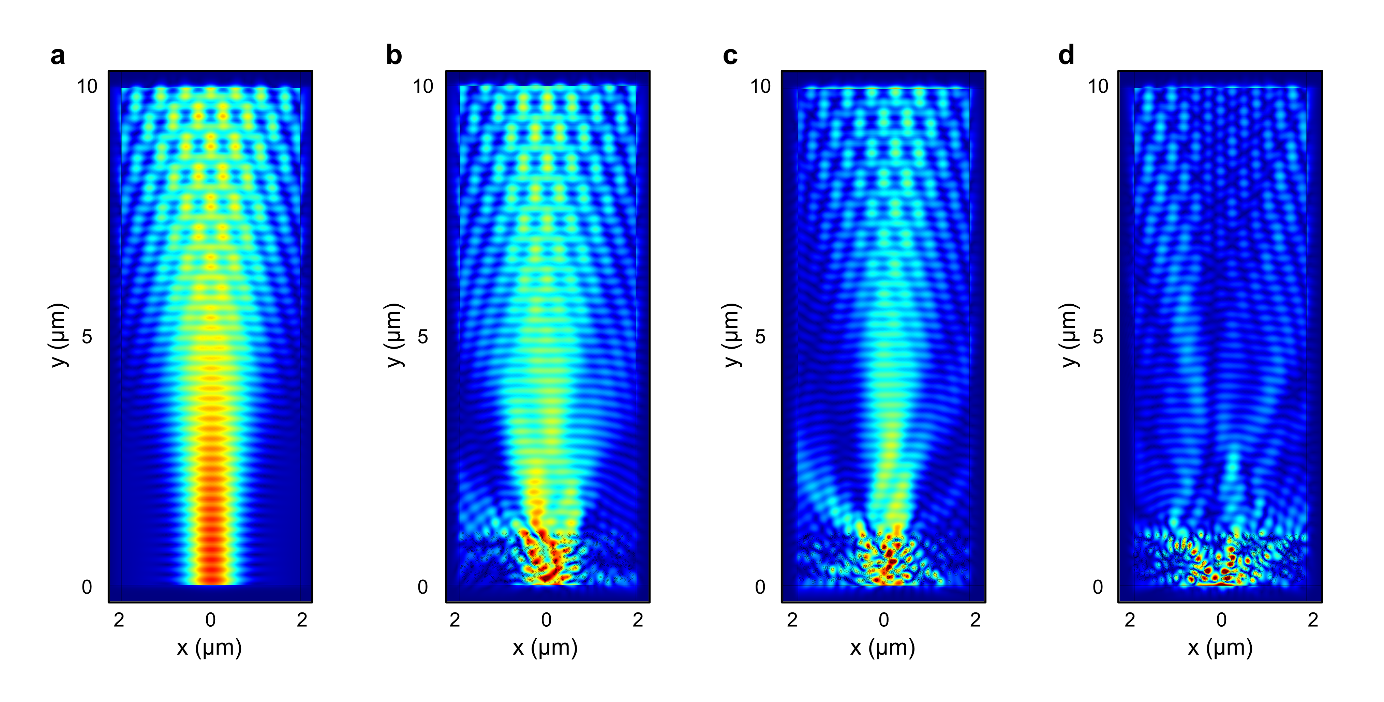


**Figure S7. Simulation results of beam scattering in residual layers with varying nanoparticle sizes. (a)** Propagation of a Gaussian beam in a homogeneous material without nanoparticles. Beam propagation in a residual layer containing TiO_2_ nanoparticles with sizes of **(b)** 30 nm, **(c)** 50 nm, and **(d)** 100 nm.


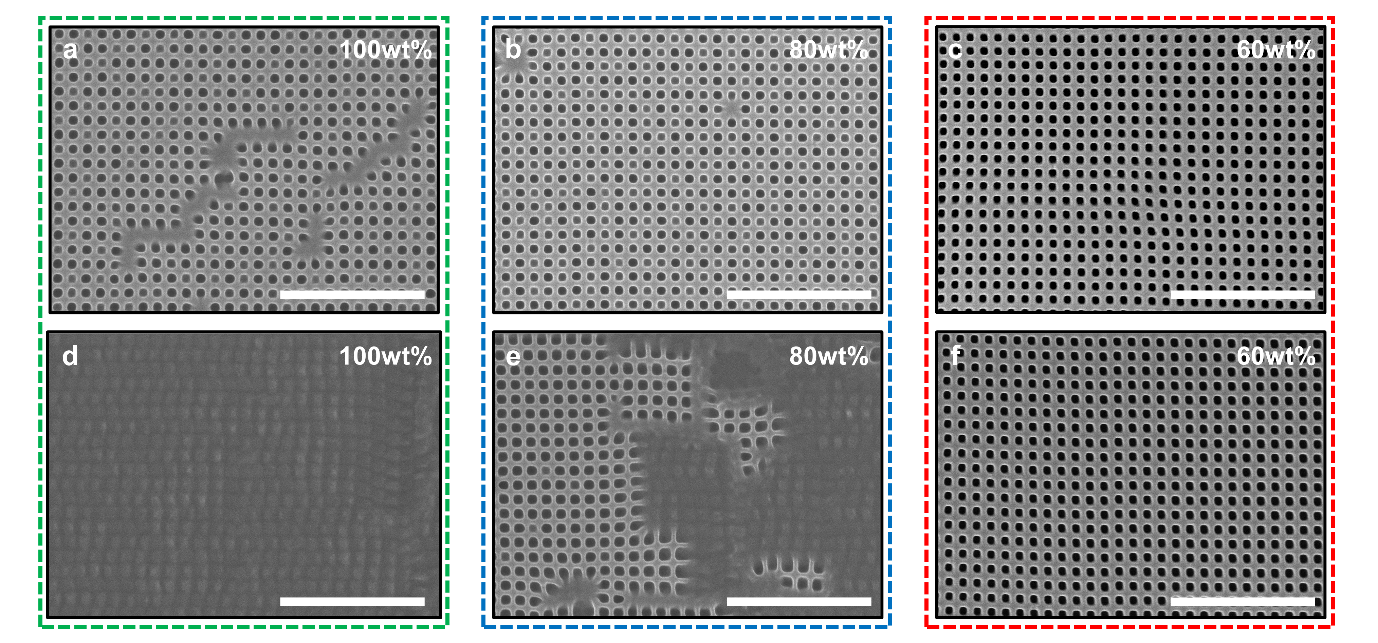


**Figure S8. Optimal weight ratio tests of toluene-diluted PDMS.** Soft mold replicated from master mold with *p*=300nm, and **(a, b, c)** *g* = 110nm, **(d, e, f)** *g* = 100nm. Replicated soft molds using various weight ratios of PDMS diluted in toluene: **(a, d)** 100%, **(b, e)** 80%, **(c, f)** 60%. All scale bars: 3 µm.


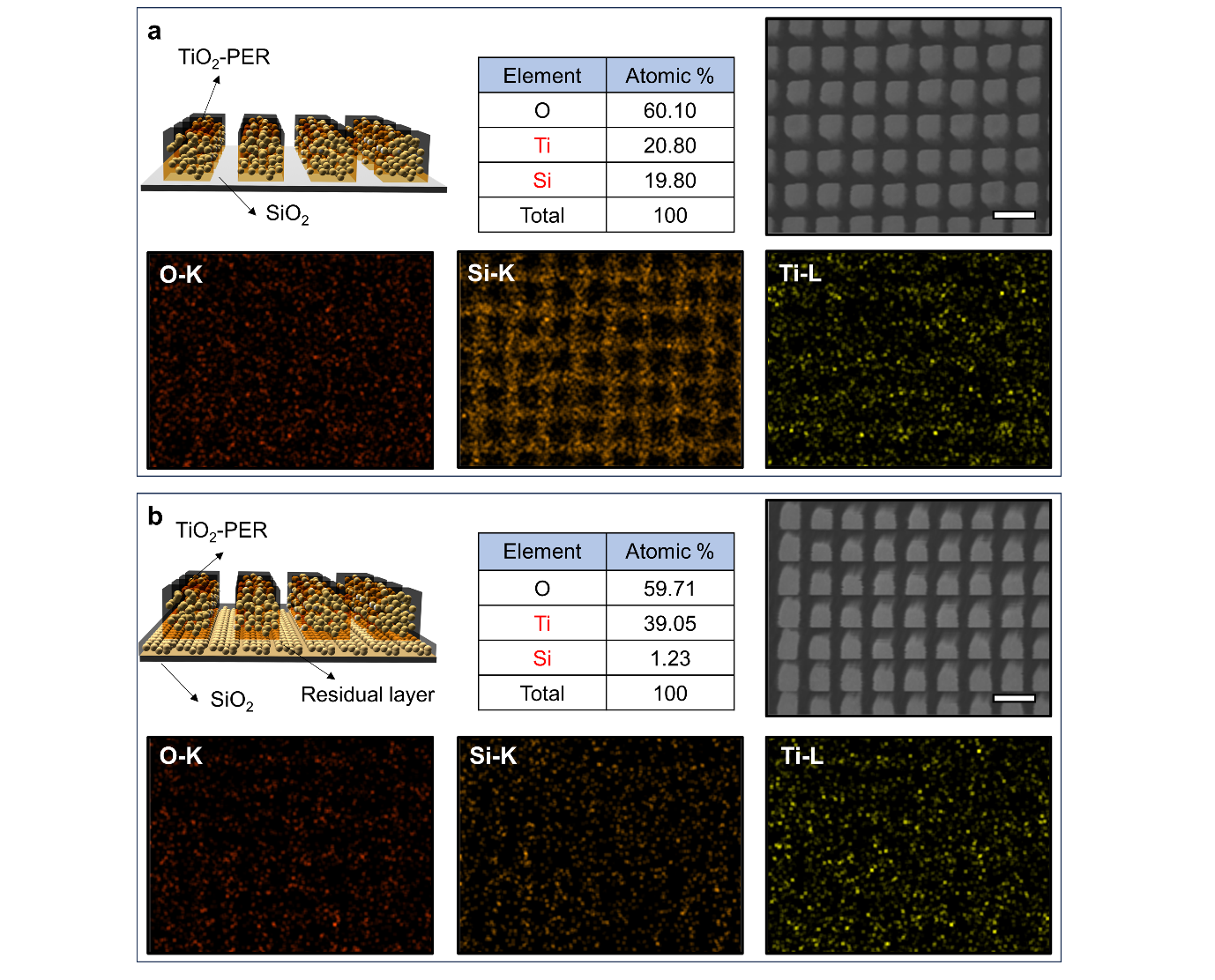


**Figure S9. Experimental validation of the residual layer removal method.** EDS analysis of **(a)** residual layer-free metasurface, and **(b)** residual layer-remaining metasurface. Scale bars: 0.5 µm.


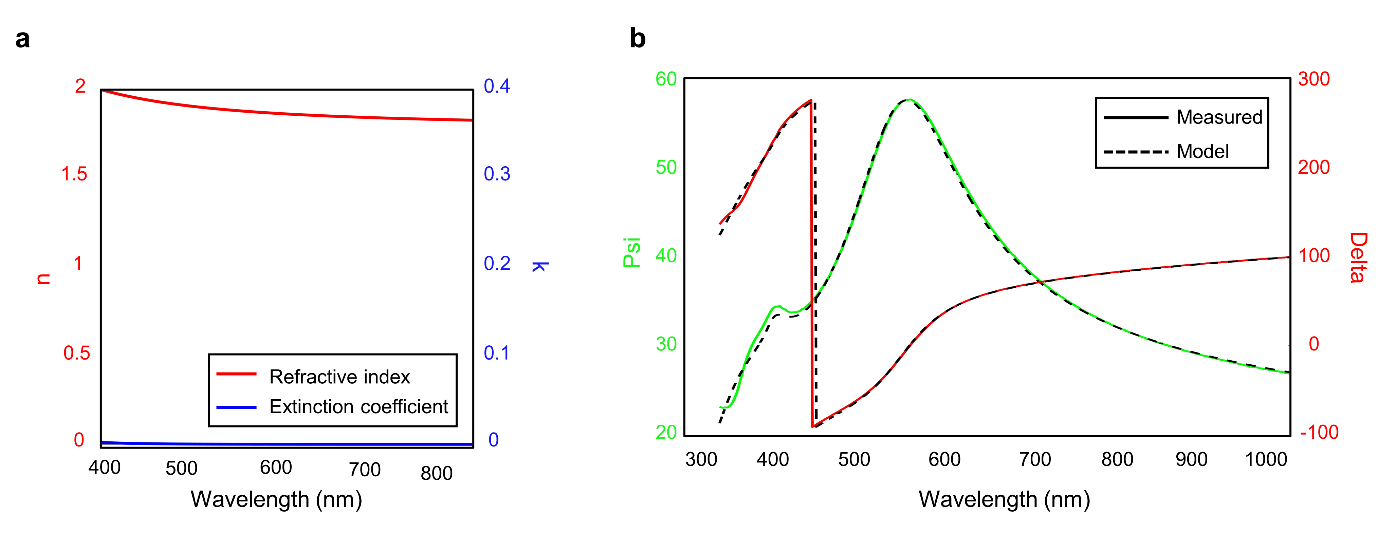


**Figure S10. Optical properties of TiO_2_-PER. (a)** Measured refractive index (*n*), and extinction coefficient (*k*) using commercial ellipsometry. **(b)** Measured amplitude ratio (Psi) and phase difference (Delta) of the 60 wt% TiO_2_-PER.


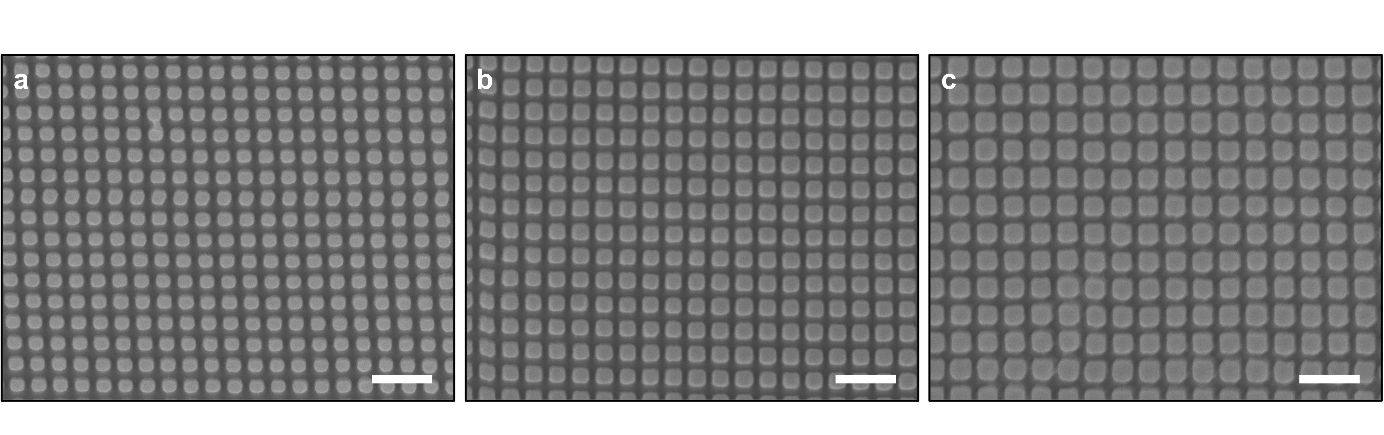


**Figure S11. SEM images of residual layer-free structural color metasurfaces.** Metasurfaces with different periods: (a) 310 nm, (b) 360 nm, and (c) 400 nm, all featuring a fixed gap of 120 nm and a height of 340 nm. All scale bars: 1 µm.


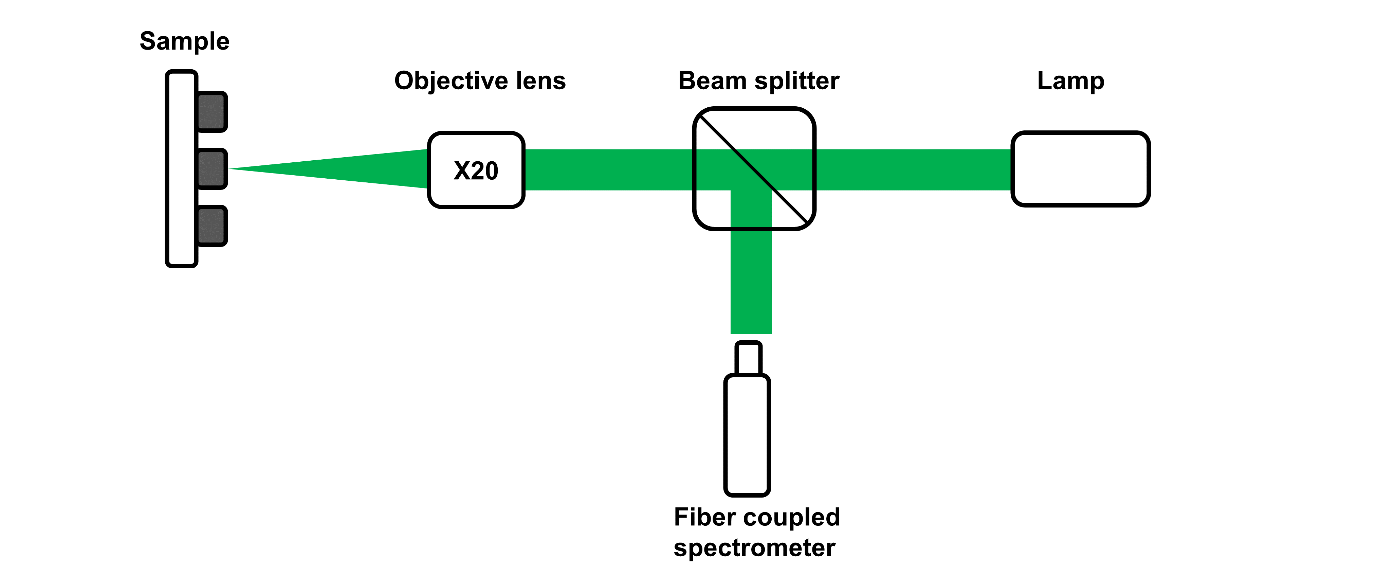


**Figure S12. Optical setup for measuring structural color metasurfaces.**


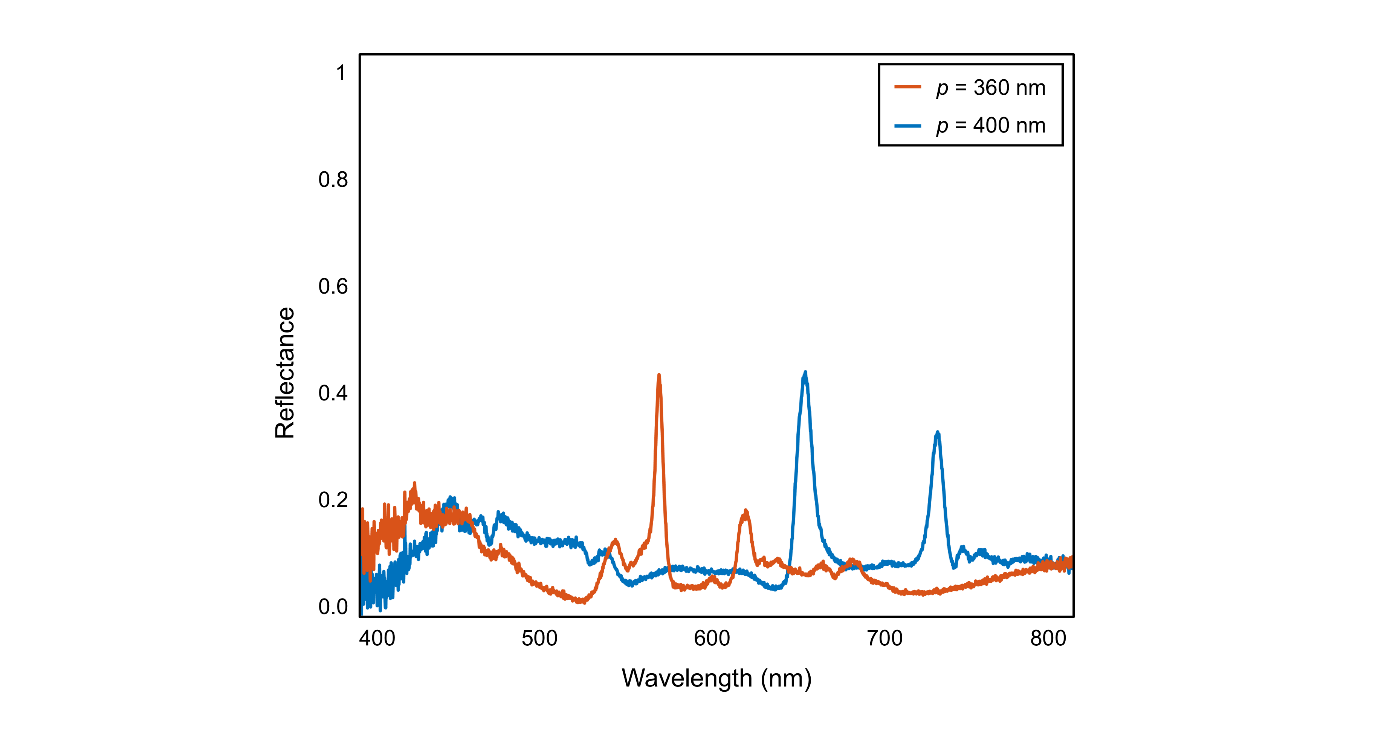


**Figure S13. Measured reflectance spectra of structural color metasurfaces with the residual layer thickness *t_residual_* = 150 nm.** The orange line represents the metasurface with a period (*p*) = 360 nm, while the blue line represents the metasurface with a period (*p*) = 400 nm.

**
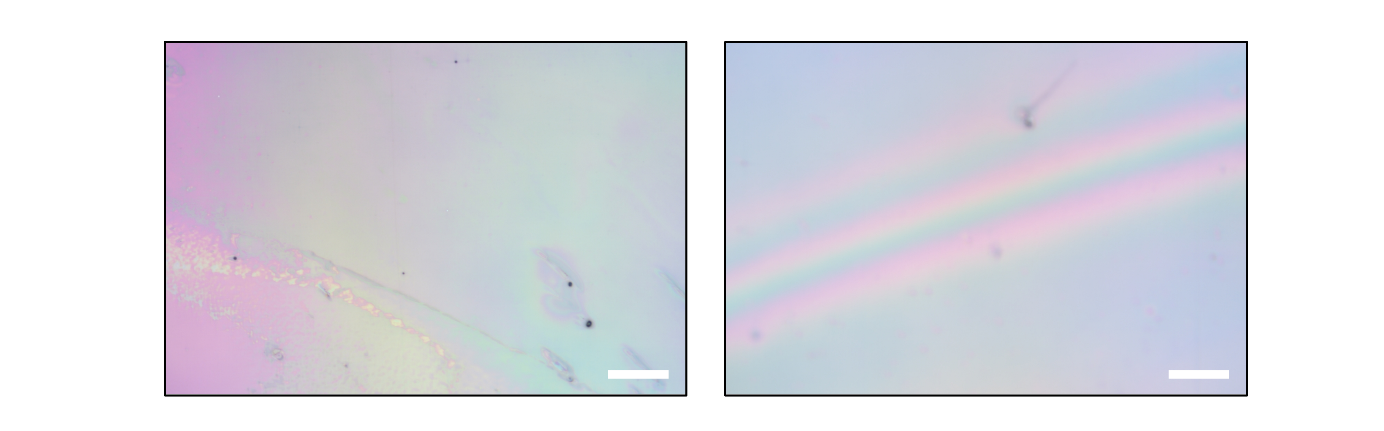
**

**Figure S14. OM images of uneven colors due to non-uniform residual layer thickness.** Scale bars: 100 µm.


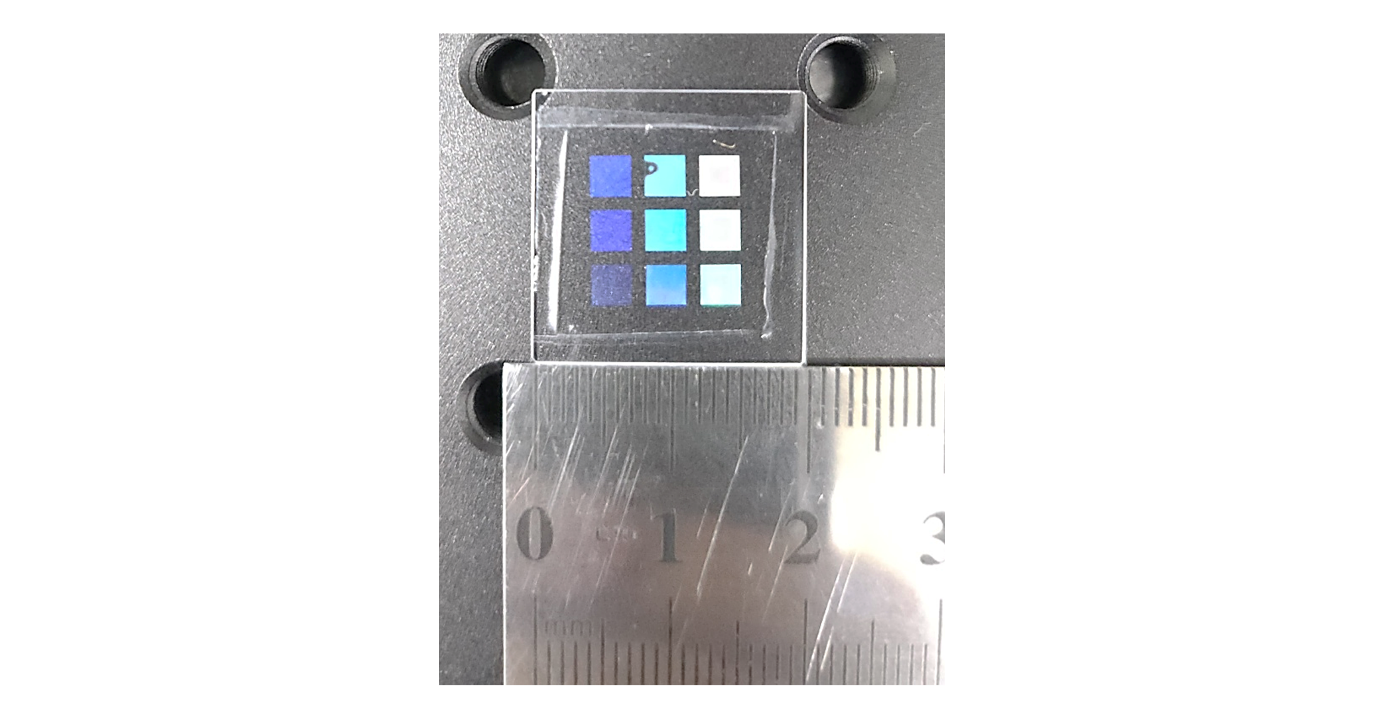


**Figure S15. Photograph of fabricated residual layer-free structural color metasurfaces with pattern sizes of 3 mm × 3 mm, respectively.**

**
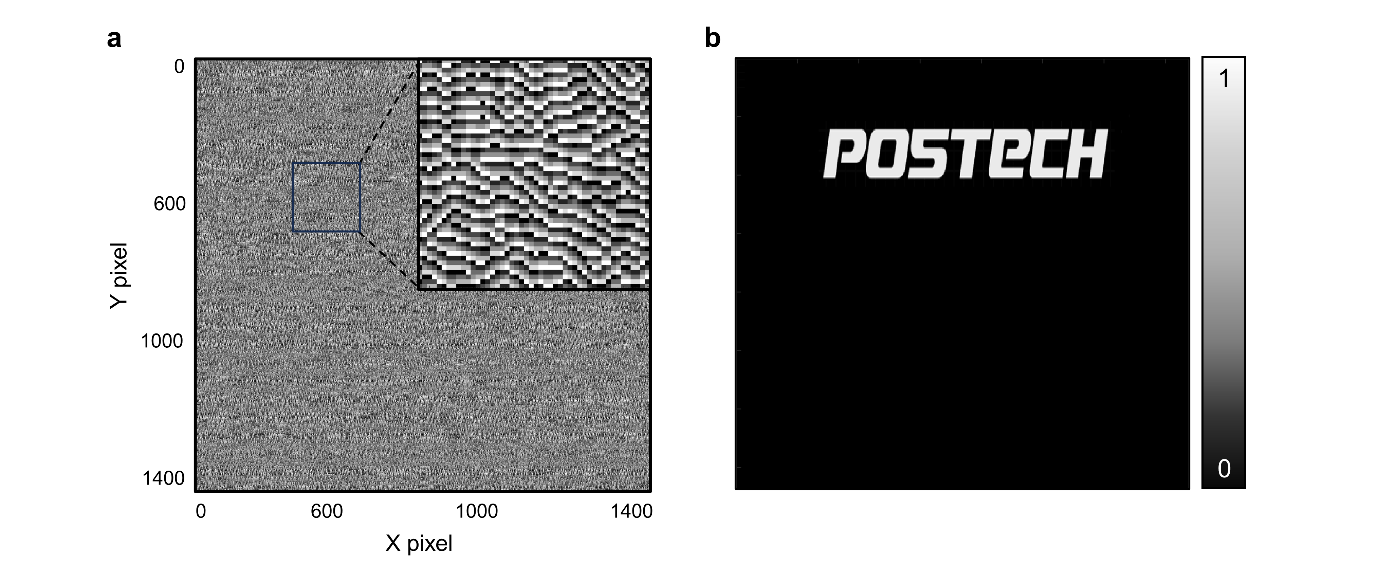
**

**Figure S16. Design of hologram metasurface.** **(a)** Phase map using computer-generated holography algorithm for a phase-only hologram metasurface. **(b)** Target image from the phase map.

**
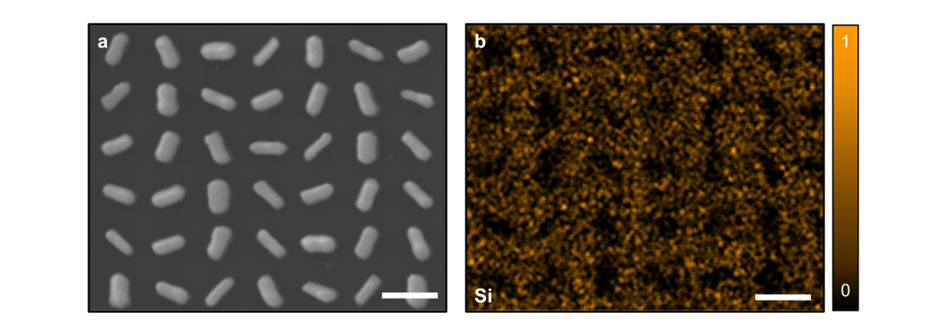
**

**Figure S17. Imprinted residual layer-free hologram metasurfaces. (a)** SEM image of an imprinted hologram metasurface without residual layer. **(h)** EDS analysis of Si element of the residual layer-free hologram metasurface. All scale bars: 0.5 µm.


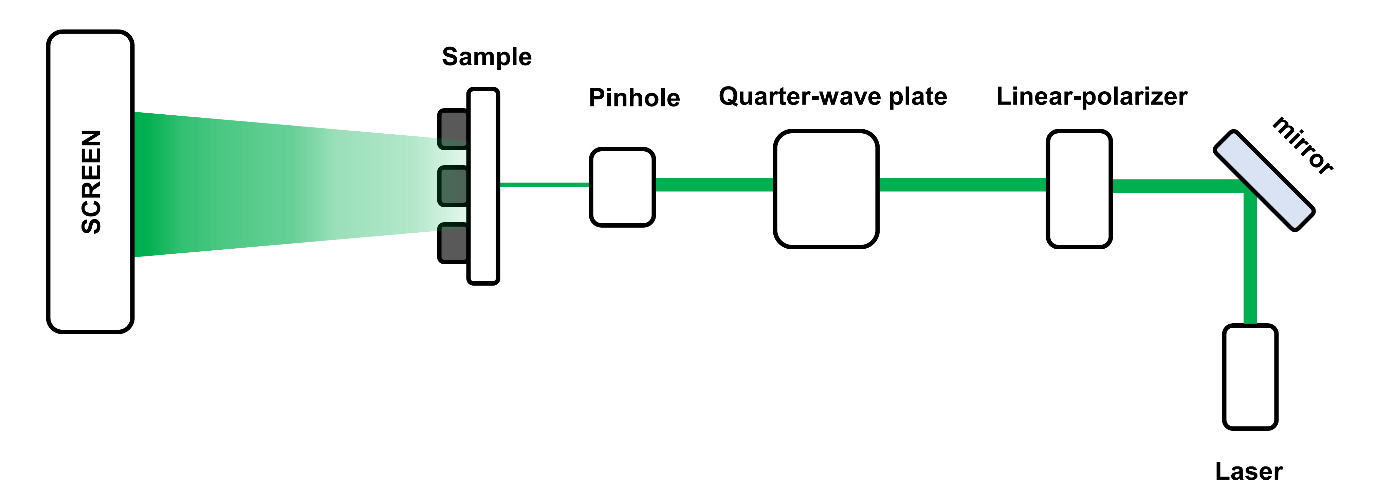


**Figure S18. Optical setup for measuring hologram metasurfaces.**

**
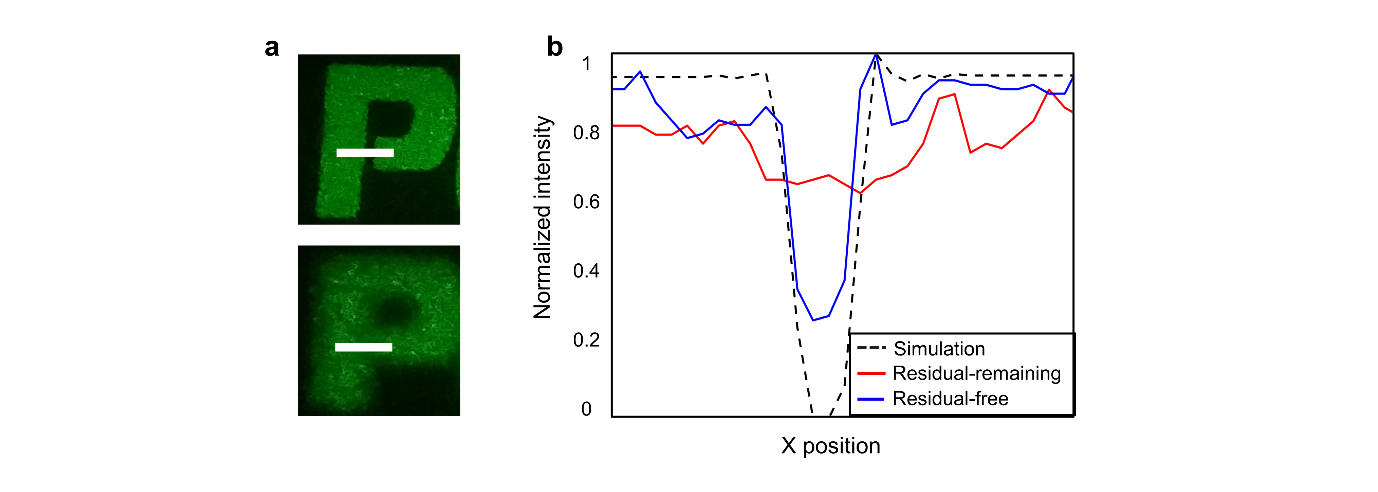
**

**Figure S19. Normalized intensity profile of hologram metasurfaces.** **(a)** Intensity profiles are analyzed across the cross-section of white line. The top image illustrates the residual layer-free hologram, while the bottom image illustrates the residual layer-remaining hologram. **(b)** Normalized intensity profile of the hologram metasurface: (red line) with a residual layer, (blue line) without a residual layer, and (black dashed) a simulattion result.


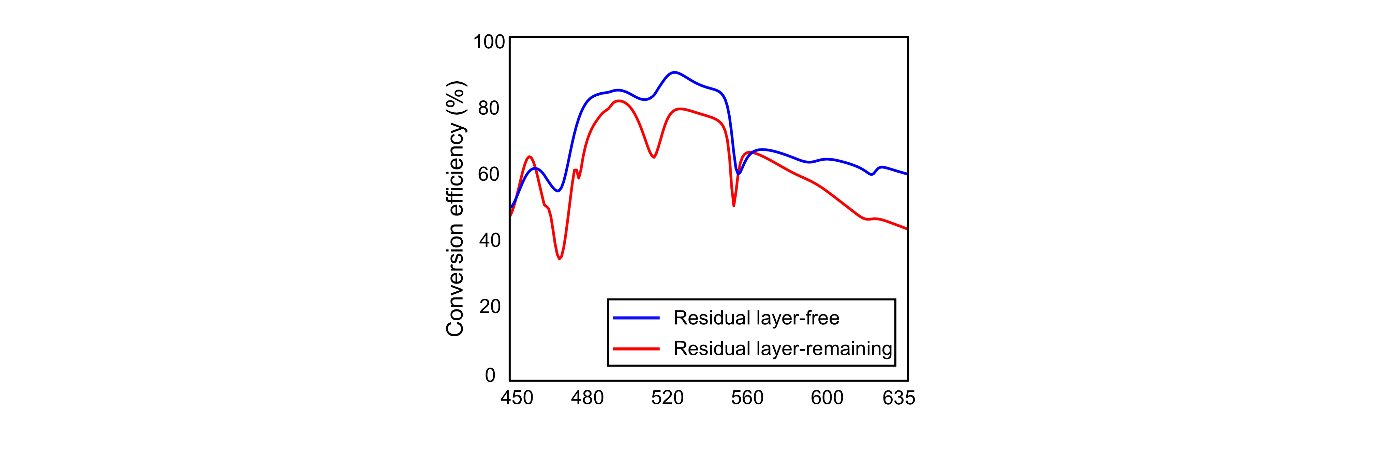


**Figure S20. *CE* of meta-atoms with and without residual layer in visible wavelengths** Red line represents a meta-atom with *tresidual* = 70 nm; blue line represents one without residual layer.

**
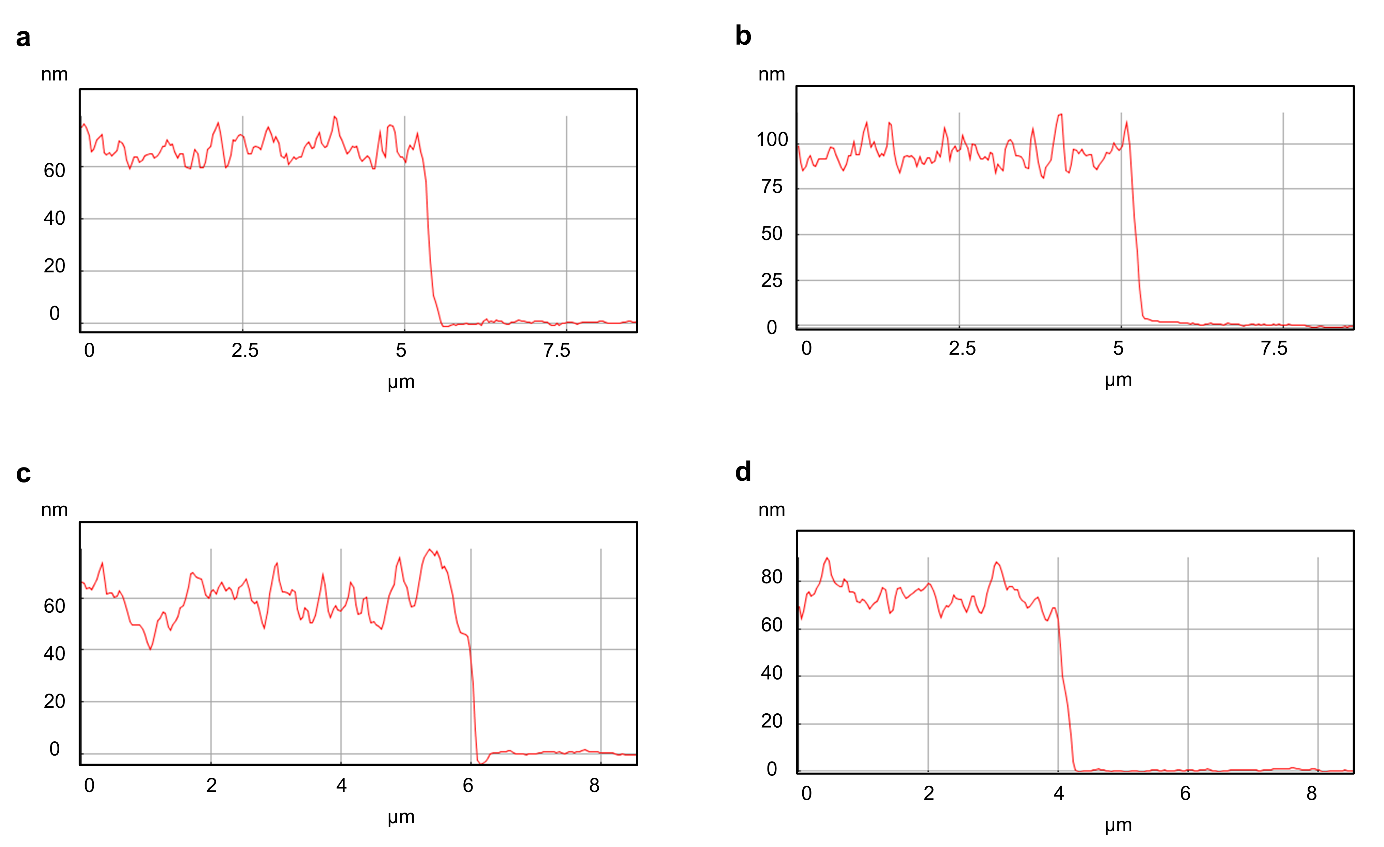
**

**Figure S21. Residual layer thicknesses measured by atomic force microscopy (AFM).** Surface z-step profiles are obtained at the boundary between the residual layer and the substrate for spin-coated samples at different revolutions per minute (RPM): (a) 2000, (b) 3000, (c) 4000, and (d) 5000.

**
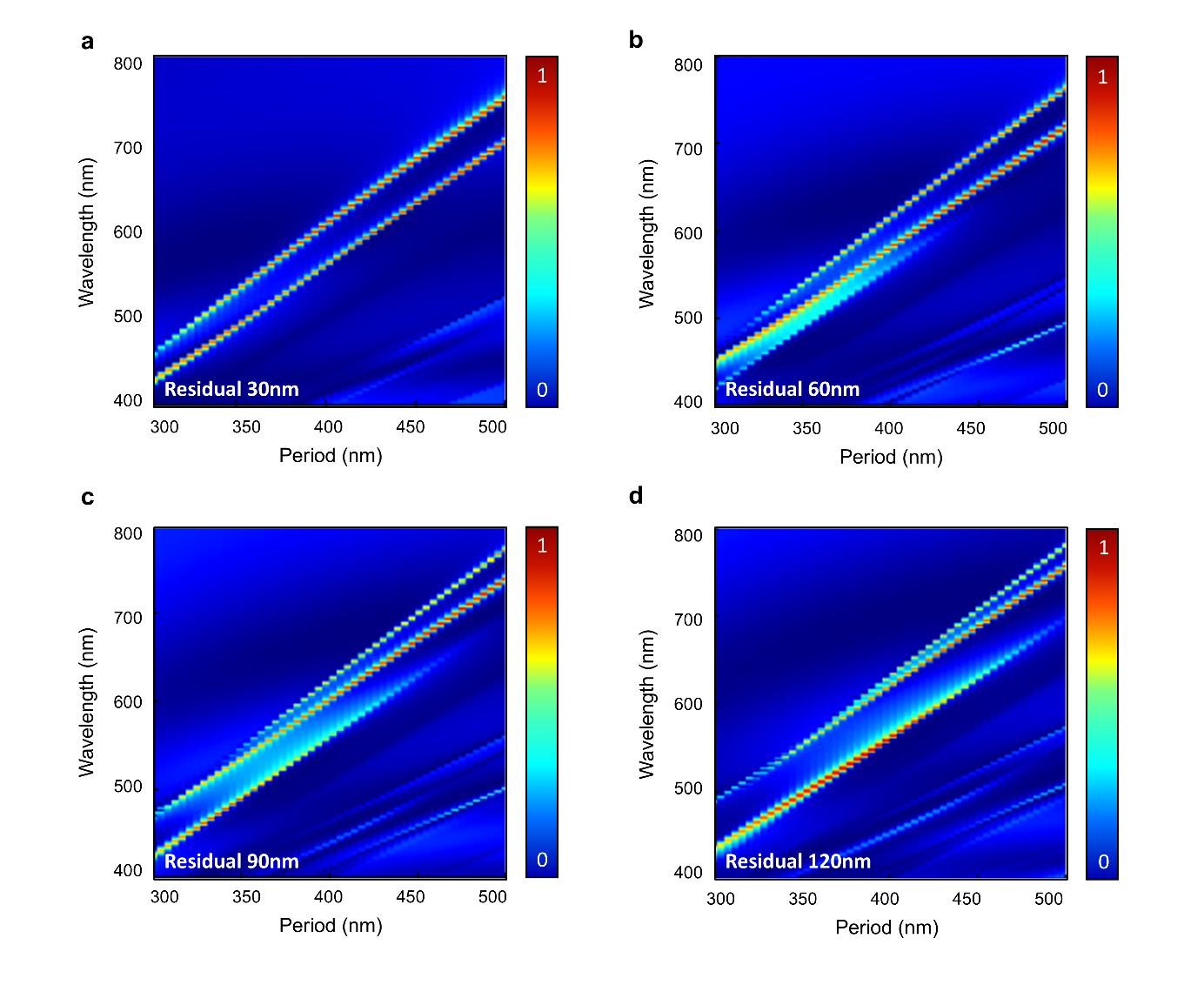
**

**Figure S22. Simulated reflectance spectra of varying resiual layer thicknesses.** Reflectance spectra of structurl color metasurfaces with a fixed *g* of 120 nm, and *t* of 340 nm for *t_residual_* of (a) 30 nm, (b) 60 nm, (c) 90 nm, and (d) 120 nm.


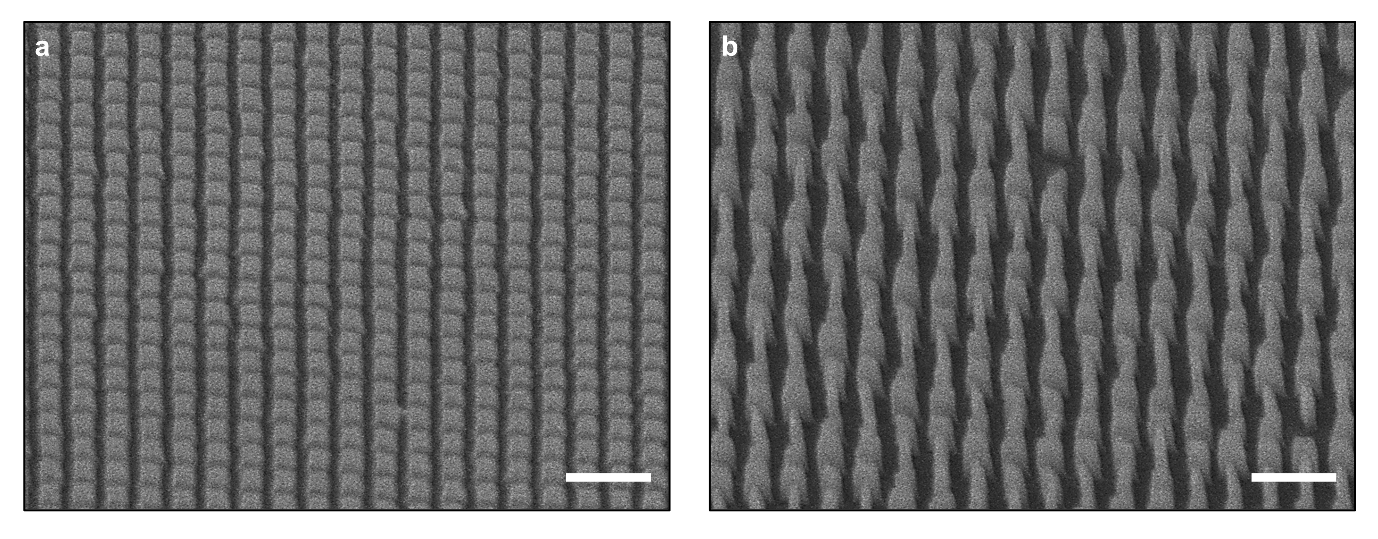


**Figure S23. 45˚ tilted SEM images of imprinted residual layer-free metasurfaces**. SEM images of **(a)** a structural color metasurface with a meta-atom thickness of *t* = 340 nm, and **(b)** a hologram metasurface with *t* = 1000 nm. All scale bars: 1 µm.
